# Lumpy Skin Disease: Insights into Molecular Pathogenesis and Control Strategies

**DOI:** 10.3390/vetsci11110561

**Published:** 2024-11-13

**Authors:** Ali Haider, Zaheer Abbas, Ahsen Taqveem, Abid Ali, Mohsin Khurshid, Rania F. El Naggar, Mohammed A. Rohaim, Muhammad Munir

**Affiliations:** 1 Department of Allied Health Sciences, The University of Lahore, Gujrat Campus, Gujrat 50700, Pakistan; 70076430@student.uol.edu.pk (A.H.); 70059604@student.uol.edu.pk (Z.A.); 2 Institute of Microbiology, Government College University Faisalabad, Faisalabad 38000, Pakistan; ahsentaqveem13@gmail.com (A.T.); mohsinkhurshid@gcuf.edu.pk (M.K.); 3 Department of Allied Health Sciences, The University of Chenab, Gujrat 50700, Pakistan; abidali@ahs.uchenab.edu.pk; 4 Department of Virology, Faculty of Veterinary Medicine, University of Sadat City, Sadat 32897, Egypt; r.f.elnaggar@lancaster.ac.uk; 5 Department of Virology, Faculty of Veterinary Medicine, Cairo University, Giza 12211, Egypt; mohammed_abdelmohsen@cu.edu.eg; 6 Division of Biomedical and Life Sciences, Faculty of Health and Medicine, Lancaster University, Lancaster LA1 4YG, UK

**Keywords:** pathogenesis, transmission, lumpy skin disease virus, disease control, livestock, vectors

## Abstract

Lumpy skin disease (LSD) is a viral infection that affects buffaloes and cattle across various regions, including both tropical and temperate climates. Intriguingly, the virus–carrying skin sores remain the primary source of infection for extended periods, exacerbated by the abundance of vectors in disease–endemic countries. Recent scientific advances have revealed the molecular aspects of LSD and offered improved vaccines and valuable antiviral targets. This review summarizes the molecular features of LSD and its effect on various livestock species. We then provide an extensive discussion on the transmission dynamics of LSD and the roles of vectors in its continued spread among livestock populations. Additionally, this review critically analyses the rationales behind, as well as the affordability and effectiveness, of current control strategies worldwide.

## Simple Summary

Lumpy skin disease (LSD) is a viral disease impacting buffaloes and cattle, which are vital to the global economy. The disease causes nodular skin lesions and sometimes affects other body systems, resulting in economic setbacks such as a lower milk yield, weight loss, fertility issues, and reduced livestock exports. Controlling LSD effectively involves vaccination, quarantine, vector management, and tracking animal movement, particularly across borders. Grasping its spread through epidemiological studies and training livestock handlers on recognizing symptoms of LSD, as well as implementing biosecurity measures, vaccination techniques, and vector management strategies, are crucial steps in managing and reducing the disease’s effects.

## 1. Introduction

Lumpy skin disease (LSD) is a viral transboundary disease with the potential to extend beyond its initial outbreak region, possibly developing into an epidemic [1,2]. The primary clinical manifestations involve the presence of nodular lesions on both the dermal layer and mucosal surfaces. These nodules frequently appear on the external regions of infected cattle, such as the neck, perineum, udder, head, and other areas [3,4]. Affected cattle often display varying degrees of edema and lameness [5]. Additionally, infected animals commonly exhibit mucosal ulcerations, an elevated body temperature, and enlarged lymph nodes [6,7].

While a substantial percentages of the affected bovine population may eventually recover after an extended period of disease, they will continue to exhibit persistent symptoms of mastitis, pneumonia, and dermal ulcerations [4,8]. Due to its highly contagious nature, the World Organization for Animal Health requires LSD to be reported as a transmissible disease. LSDV has the ability to spread in various ways, including secondary contact among animals through vectors, semen, natural suckling, biting insects, and iatrogenic transmission [9,10]. However, several studies have verified that the transmission of the disease through direct contact is challenging [11,12].

The initial documentation of LSD was in Zambia, South Africa, in 1929 (formerly known as Rhodesia in 1932). In recent decades, LSDV has spread rapidly across the Middle East, North Africa, Asia, and other regions, significantly impacting cattle and water buffalo. Cattle breeds with thinner hides, such as the Holstei–Friesian and Jersey breeds, are more susceptible, while Bos indicus cattle have thicker skin due to selective breeding, and varieties like Afrikander exhibit less pronounced symptoms of the disease [13]. Although the effectiveness of preventive measures against LSD is limited, measures such as enforcing movement restrictions on infected cattle, establishing quarantine protocols, and euthanizing infected cattle are strongly recommended [14]. On the other hand, the effective management and mitigation of LSD in countries like Bulgaria, Montenegro, Greece, FYROM, Albania, Ethiopia, and Serbia rely heavily on the widespread adoption of immunization strategies [15,16]. Due to the intricate immune evasion mechanisms exhibited by LSDV, the development of safe and efficient vaccines against it remains elusive [17].

Cross–protection between sheeppox virus (SPPV) and goatpox virus (GTPV) is possible due to their antigenic similarities with LSDV. Consequently, vaccines developed for SPPV and GTPV can be employed as preventive measures against LSD. However, it is important to note that SPPV and GTPV vaccines carry inherent risks as they are live–attenuated and developed from field–isolated strains [18,19]. Therefore, their use is not advisable in regions where the disease is absent. The diagnostic tools for LSD primarily target the nucleic acid sequence of LSDV or associated antigens and antibodies [20]; however, different diagnostic techniques exhibit varying degrees of reliability depending on specific factors such as the stage of infection, the type of sample collected, and the prevalence of the disease in the region. For instance, polymerase chain reaction (PCR) tests are highly sensitive and can detect low levels of the virus in samples from infected animals, making them effective during the early stages of infection. In contrast, serological tests, which detect antibodies against the virus, may be less reliable in the early stages when antibody levels are still low, but they can provide a better indication of past exposure to the virus in regions where LSD has been endemic [21].

## 2. Etiology

**Figure 1:**
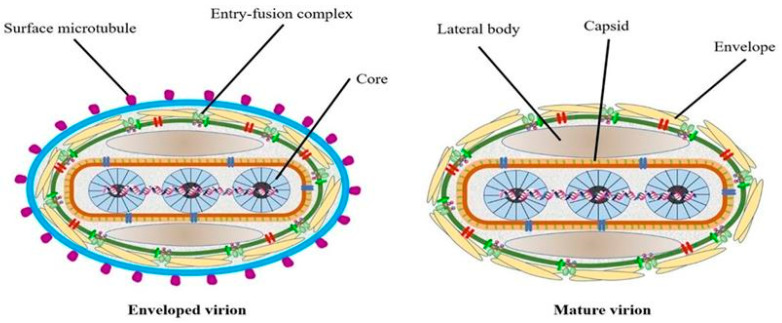
A diagram illustrating the predicted structure of LSDV. The mature virion of LSDV (MV) can sometimes be found enclosed in a lipid membrane that originates from the endoplasmic reticulum (EV). The virus is enveloped, with an entry–fusion complex on its surface. The virus is composed of a lateral body, capsid, and core. The EV’s surface is covered in numerous microtubules [17].

The lumpy skin disease virus (LSDV), which causes lumpy skin disease (LSD), belongs to the genus *Capripoxvirus* within the subfamily *Chordopoxvirinae* of the family *Poxviridae*. It is characterized by a large, linear, double–stranded DNA genome that spans approximately 151 kb and features a core coding region flanked by inverted terminal repeat sequences [22]. Although the precise structural diagram of LSDV particles remains undetermined, a predictive model based on known poxvirus structures has been proposed, as shown in Figure 1 [17]. The *Capripoxvirus* genus, encompassing LSDV, is notably virulent in domestic ruminants across Africa and Asia. LSDV exhibits a 97% nucleotide sequence homology with GTPV, indicating a shared evolutionary origin and subsequent genetic recombination among various hosts [23]. The inclusion bodies of LSDV bear a resemblance to those found in other members of the *Poxviridae* family, a similarity confirmed by recent electron microscopy studies [24,25]. LSDV possesses 156 potential genes and exhibits physical similarities with other poxviruses, though it does not induce blood clotting. LSDV replicates in several primary cell types, including lamb and calf kidney cells, sheep embryonic kidney cells, and chicken embryo fibroblasts. However, its growth is limited in African green monkey kidney (Vero) cells [26]. After multiple passages, LSDV can adapt to Vero cells, leading to an increase in viral titers, which is important for vaccine production and the creation of recombinant viruses. Additionally, LSDV is capable of replicating on the chorioallantoic membrane of chicken embryos, where it forms pock–like lesions without causing embryonic mortality [27]. Chinese researchers have noted elevated virus titers in primary cow testicular cells, indicating potential methods for vaccine development [28]. Additionally, LSDV’s double–stranded DNA structure exhibits heat stability, with complete inactivation occurring at 56 °C [29].

## 3. Transmission of Lumpy Skin Disease

### 3.1. Non–Vector Transmission

Direct contact does not significantly facilitate the transmission of LSDV, although there are limited experimental data available. Initial observations in South Africa indicated that transmission of LSDV through direct contact occurred at low rates and efficiency [30,31], which supported by the occurrence of LSD outbreaks outside the optimal temperature range for insect activity [32]. In contrast, during cooler temperatures or reduced rainfall, fewer insects are present, leading to a decrease in LSDV infections [33,34], Circumstantial evidence further suggests that vectors can play a role in the spread of LSD, as restricting cattle movement did not effectively control the outbreaks [34]. However, risks arise when animals share water holes or when new animals are introduced [34]. Previous research involved seven trials in which one non–infected cow was housed with two LSDV–infected animals for a month to study the direct–contact transmission model of the disease [35]. Despite this close contact, neither directly inoculated nor sentinel contact animals displayed clinical symptoms or serum–neutralizing antibodies [35]. Furthermore, six out of seven animals exhibited no evidence of delayed–type hypersensitivity after being challenged with virulent LSDV [35]. However, it is important to note that due to the small number of in–contact animals, drawing conclusive findings from this research is challenging [35]. Additionally, the experiment used a single animal, with no confirmation as to whether lesions developed in the nasal or oral mucosal membranes, or if the animal secreted the virus in its nasal discharge and saliva. Another study, which utilized mathematical modeling on an Israeli dairy farm in 2006, found no correlation between cattle density and infection rates, suggesting that vectors like blood–sucking insects, rather than direct contact, were responsible for LSD transmission. Moreover, any animal exhibiting severe clinical signs was promptly removed from the herd, potentially affecting the impact of reduced animal–to–animal contact [11]. In addition, the cattle used to infect naïve animals must exhibit numerous skin bumps and ulcerative sores in their mouths and noses to cause field outbreaks. Under these conditions, the animals can release sufficient contagious viruses in their nasal discharge and saliva to cause significant concern [11]. Similarly, sheeppox and goatpox viruses can spread through exposure to infectious virus particles in aerosols or droplets [36]. Moreover, saliva or nasal discharge from infected cattle could contaminate feed or water troughs, thereby indirectly facilitating LSDV transmission [33]. A study found that virus levels remained low in the saliva and mucus 12–18 days after infection, even in animals with moderate LSD, showing nodules covering 25% of their skin. Notably, virus loads in the mouth and nose mucous membranes were comparable to those in cutaneous lesions [37]. Another study identified erosions and ulcerations in the nose, larynx, mouth, trachea, and pharynx of highly infected animals that might release virus particles, and the droplets from the animals’ mouths and noses could potentially harbor contagious viruses for an extended period [38]. The results obtained from nose swabs or saliva samples demonstrated comparability to skin samples, as indicated by field experience [39]. Consequently, even though the presence of low virus titers in nasal or other discharges might mitigate the risk of contact–based transmission [33], it is crucial to revisit the direct mechanism underlying LSDV transmission. Recent evidence supports the possibility of LSDV transmission during pregnancy, suggesting that infection might spread from mother to calf through milk or sores on her teats and udder [40]. A more recent study detected active virus in bovine semen at 42 dpi, and virus DNA up to 159 days post–infection [41]. Both natural mating and artificial insemination pose the risk of an epidemic, as contaminated bovine semen has been shown to facilitate transmission [42,43]. Interestingly, a similar vaccination approach successfully eliminated a virus present in sperm, although this was not the same virus used in the vaccination itself [43].

Researchers found that intradermal LSDV injection caused widespread disease in less than 20% of calves, mostly resulting in localized disease [12]. In contrast, intravenous injection led to widespread disease in 70% of the animals [12]. These findings emphasize that LSDV can be injected into the bloodstream, similar to transmission via blood–feeding insects. Moreover, contaminated vaccination needles could potentially spread LSDV within a herd [44]. The intravenous and subcutaneous experimental administration of LSDV in cattle may not always induce clinical disease, leading to challenges in evaluating iatrogenic transmission and potentially exacerbating the disease [44]. This highlights the need for further research on transmission, including the use of highly virulent LSDV strains and advanced molecular techniques.

### 3.2. Flies–Based Transmission

Fowlpox, myxoma, and swinepox viruses have the potential to be mechanically transmitted by arthropods [45]. For instance, rabbit (Shope) fibroma virus can be mechanically spread by fleas, other biting arthropods, and mosquitoes. The effectiveness of these vectors in transmitting these viruses depends on factors such as vector abundance, host availability, feeding activity, bite frequency, and the likelihood of vector involvement [46]. LSDV is mainly transmitted mechanically, but field observations suggest the possibility of biological transmission by *Culicoides midges*, which warrants further investigation [47]. During the 2014–2015 outbreaks in Turkey, it was observed that non–engorged female *Culicoides* punctatus from affected farms tested positive for LSDV DNA, even though there was no evidence of recent bovine feeding [48]. Unlike biological transmission, mechanical transmission is not limited to a specific vector species [49]. Any local vector species that prefer cattle and frequently change hosts could potentially carry the infectious virus in their mouthparts [49]. Understanding the biology, feeding preferences, and behaviors of local arthropod species is crucial in this context. High–titer virulent virus injections in experimental LSD cattle infections resulted in severe illness in only 70%, suggesting that multiple vector bites are needed for transmission; however, the impact of arthropod saliva on host immunity and its role in reducing viral transmission remains unstudied [35].

Culex mosquitoes, like *Culex quinquefasciatus*, may transmit LSDV after feeding on infected hosts multiple times, though they typically avoid returning to the initial host for subsequent bites, potentially increasing transmission risk [49]. Species like *Say and Anopheles stephensi Liston* tested positive for LSDV but did not transmit the virus under laboratory conditions [50]. Mosquitoes and sandflies can introduce LSDV intravenously by feeding on narrow blood vessels within days after consuming LSDV–rich skin lesions [51]. In addition, various mosquito species may serve as mechanical vectors for LSDV in regions with ongoing outbreaks, potentially transmitting the virus by feeding on multiple hosts [51]. Certain mosquito species can carry the virus for extended periods and inoculate multiple times, but the transmission efficiency depends on the virus titer in their blood [52]. Researchers have postulated that insects such as horn flies (*Haematobia irritans*), lice (*Hippoboscidae*), and horse flies (*Tabanidae*) may contribute to the disease spread [51]. Moreover, infected sheep head flies have been examined and used to isolate SPPV, while biting lice (*Mallophaga* spp.), sucking lice (*Damalinia* spp.), and midges (*Culicoides nubeculosus*) were found to be ineffective in terms of virus transmission [52]. The systematic monitoring of vector species abundance and activity could enhance the risk assessment process, and increased research on LSDV vectors in the northern hemisphere holds promise for advancing our understanding of vector–mediated transmission in the future [52].

### 3.3. Tick–Based Transmission

A virus’s ability to tolerate histolysis in tick tissues and the susceptibility of tick cells to infection determine its survival in tick vectors [53]. Ticks can mechanically transmit the virus if they feed multiple times and switch hosts. For example, Fowlpox virus can be mechanically transmitted via ticks [54,55]. Female ticks typically feed on one host per life–cycle stage, while adult males of certain hard tick species often consume multiple small blood meals, primarily in the morning; nonetheless, females may feed on multiple hosts if the current host dies or if feeding is interrupted by extensive grooming [56].

Male ticks of *Rhipicephalus appendiculatus* and *Amblyomma hebraeum* may transmit LSDV mechanically, with the virus detected in tick saliva after feeding on infected cattle and observed across various tick tissues [57]. The *Rhipicephalus decoloratus* tick, known for its host specificity, transmits LSDV from infected cattle to naive ones through the vertical transmission of the virus from females to their eggs, suggesting a potential mechanical mode of transmission similar to that observed in *Hyalomma truncatum* transmitting the Crimean–Congo hemorrhagic fever virus [58]. Males become infected by feeding on the skin of infected animals and subsequently transmit the virus to females during copulation, introducing the virus from their semen sack into the female’s vaginal apertures using their mouthparts [59]. After exposure to sub–zero temperatures typical of winter, ticks are capable of transmitting LSDV transovarially [60]. Recent studies indicate that *Amblyomma ringed* ticks may transmit the virus. Mature female *Rhipicephalus annulatus* ticks oviposited on LSDV–infected calves and live virus was identified from hatchlings on chicken egg membranes, displaying raised, circumscribed, firm lesions accompanied by superficial lymph node swelling [61].

Ticks have a longer lifespan in central and southern Africa compared to the Middle East because of variations in environmental factors [62]. The transmission of LSDV can also occur through the sharing of pastures by wild and domesticated ruminants, particularly in communal grazing areas [63]. Although further research is necessary to validate these preliminary findings, it appear s that ixodid ticks might have played a role as vectors or reservoirs for LSDV during the outbreaks in 2015 [64]. Monitoring in Bulgaria found LSDV DNA in *Hyalomma marginatum* (female) and *Rhipicephalus bursa* (male and female) [65]. The intensified research on potential arthropod vectors for LSDV following the initial outbreaks in Europe underscores the need for further experimental investigation to comprehensively understand the vector capability and the potential role of ticks as reservoirs of LSDV in northern climates.

## 4. Pathogenesis

LSDV infection initiates a cascade of events including viral replication, viremia, fever, cutaneous localization, and nodule formation. Experimental studies following intradermal viral injection outlined specific timelines for various outcomes: between 4 and 7 days post–infection (DPI), nodules or plaques of 1 to 3 cm appeared at the inoculation site; between 6 and 18 DPI, viral shedding occurred through oral and nasal routes, along with viremia; between 7 and 19 DPI, regional lymphadenopathy and generalized skin nodules developed; and 42 days after fever onset, the virus was detected in semen [66]. Virus replication within macrophages, endothelial cells, pericytes, and fibroblasts resulted in vasculitis and lymphangitis [67,68]. Due to humoral immune deficiency, lactating cows, newborn calves, and underweight animals are more susceptible to natural infections, while animals that survive after virus exposure develop lifelong immunity [44,69,70]. Maternal antibodies provide passive immunity to calves, which can help protect them from infection during the first few months of life. However, once a calf is infected with LSDV, these maternal antibodies can still play a role in modulating the disease’s severity. While they do not prevent the infection itself, maternal antibodies may help reduce the intensity of clinical symptoms and support the calf’s immune response in combating the virus. This protective effect is particularly important in young animals, as their immune systems are still developing. Thus, while maternal antibodies do not prevent infection, they can mitigate the impact of the disease in already infected calves [71,72,73]. Infected animals with LSDV typically recover completely, and there are no documented carriers of the virus. Understanding the evolution and clinical manifestations of LSDV includes tracing the progression from the initial signs to subsequent symptoms [44].

LSDV infections display a diverse array of clinical manifestations, including both temporary and chronic subclinical infections, and death in severe cases [74,75]. The virus’s incubation period after successful transmission to the host varies from 7 to 28 days [17,74,75]. LSD initially manifests with fever (40 °C to 41.5 °C), depression, decreased appetite, and reduced physical activity. Within 5–7 days, numerous well–defined skin lesions (nodules) appear, ranging from 2 to 7 cm in diameter. These lesions, characterized by flat–topped papules and nodules, affect the dermis and epidermis, occasionally extending to the hypodermis and rarely to the adjacent striated muscle [76]. LSDV can persist in various reservoirs, including skin lesions, blood, scabs, oral and nasal secretions, semen, and sometimes in animal skin without apparent symptoms. It is widely distributed across several tissues, with painful nodules initially appearing near the head, mouth, nose, and eyes, then spreading to the cervical region, extremities, perineum, mammary gland, and reproductive organs [77]. During the initial phases of viral infection, these nodules exhibit a grayish–white internal coloration and may secrete serum. Around 14 days post–infection, nodules may develop a central core of necrotic material known as the “sit–fast” [78]. These necrotic cores provide an opportunity for secondary bacterial infection. As ulcers form in the infected nodules, LSDV can invade various body fluids, such as saliva, nasal and ocular secretions, and vaginal discharge [17,79]. Consequently, infected bovines often show significant emaciation and fragility, leading to prolonged productivity losses and potential skin damage [80,81].

Recent studies revealed that infected animals exhibit pathological changes in several tissues and organs, including mastitis, orchitis, necrotic hepatitis, lymphadenitis, and extensive vasculitis [82]. Symptoms can include rhinitis and nasal discharge, which initially is serious and later becomes mucopurulent. Animals may also display tracheitis, cardiac impairment, and other pathological irregularities, contributing to the severity of LSDV infection [83]. A clinical study revealed that LSDV infection disrupts the balance between oxidation and antioxidation in cattle, leading to elevated pro–inflammatory cytokines and adverse effects on health, including organ dysfunction and hypophosphatemia due to metabolite accumulation in the heart, liver, and kidneys [84]. This disorder exacerbates hemolytic anemia symptoms. Experimental findings show pancytopenia, hyperproteinemia, hyperkalemia, hyperchloremia, and lower creatinine levels in infected animals, providing valuable markers for evaluating the prognosis, severity, and effectiveness of LSD management [85]. Therefore, these markers serve as valuable tools for evaluating the prognosis, severity, and effectiveness of LSD management or control, and can allow for particular attention to be paid to the increased susceptibility of young calves, lactating mothers, and underweight animals to LSDV infection, possibly due to their compromised immune systems. Additionally, numerous reports have highlighted cases of abortions in pregnant cattle and multiple skin lesions in aborted fetuses, as well as lifelong immunity in animals that recover from the disease [86]. Calves born to infected mothers acquire maternal antibodies that offer protection against LSDV for about six months, while animals that successfully combat LSD infection clear the viral load entirely and do not act as carriers for LSDV [87].

## 5. Spatial Distribution

**Figure 2:**
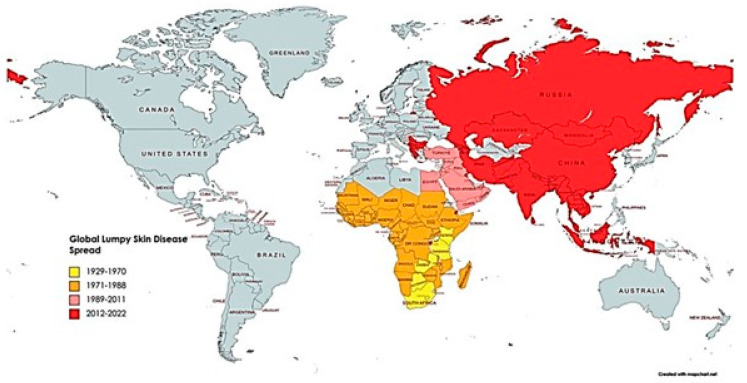
Lumpy skin disease’s prevalence worldwide and over time from 1929 to 2022. The impacted countries are shown in yellow between 1929 and 1970, orange between 1971 and 1988, pink between 1989 and 2011, and red between 2012 and 2022 [77].

LSDV was first detected in Zambia in 1929 and has since been reported in different regions across African countries as shown in Figure 2 [77]. The disease has been documented in multiple countries, including Lebanon, Iraq, Saudi Arabia, Israel, Turkey, Jordan, and Iran [88,89,90,91,92]. Since 2015, the spread of LSD has extended in various geographical regions, including Azerbaijan, Greece, Serbia, Bulgaria, Russia, Albania, Kosovo, Armenia, and Montenegro [93,94,95,96,97]. Consequently, it is essential to consider the increased risk of disease transmission to other regions of Europe and Asia [93,94,95,96,97]. During the 2012–2013 LSD outbreak in Israel, a study evaluated the efficacy of two vaccines against LSDV [98]. The results indicated that the Neethling vaccine was more effective compared to RM65, despite a modest incidence of Neethling–related sickness. Neethling vaccination showed a lower risk of LSD morbidity and severe illness, underscoring its higher effectiveness in controlling the disease [98].

### 5.1. Incidence and Death Rates

The OIE reported no specific timeframe for the development of LSDV infection in natural environments as of 2018. Morbidity rates ranged from 5% to 45%, with mortality rates typically below 10% but occasionally reaching up to 40% [70,99]. For example, during LSD outbreaks in Greece [96] and Turkey [100], the incidence of disease and death rates was 8.7% and 0.4% and 12.3% and 6.4%, respectively [96,100]. The severity of LSD is influenced by factors such as animal age, breed, immune status, and production period [101].

### 5.2. Factors Contributing to Increased Risk of LSD

**Figure 3:**
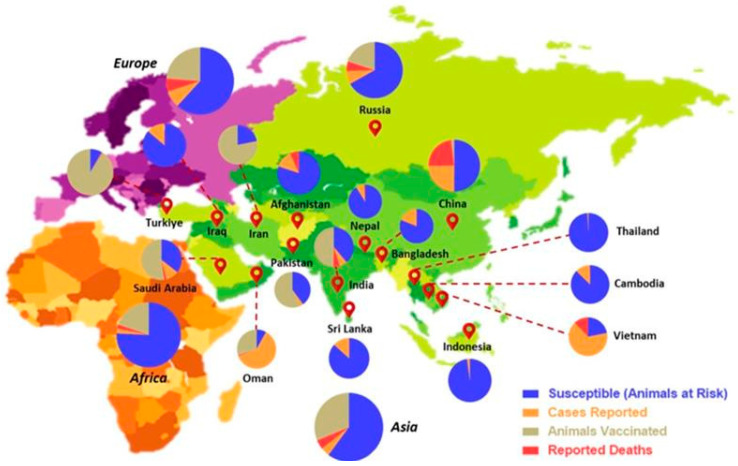
The geographical distribution of LSDV infections across various countries over the past five years [83]. This represents a comprehensive geographical overview of LSD outbreaks reported in the past five years at the global level. The statistics are quantified based on the categorization made by WOAH with respect to LSDV infections (reported cases; deaths; susceptible and vaccinated animals).

Several risk factors contribute to the spread of LSD, including climatic conditions that favor vector replication, the introduction of new animals, herd size, vector populations, proximity to water bodies, migration patterns, the transport of infected animals, and shared pasture and water sources [102]. Additionally, the speed and direction of air flows might influence the virus’s spread. LSD can affect cattle of all ages and breeds, regardless of their sex. Furthermore, factors associated with LSDV seropositivity include age, gender, management practices, average yearly precipitation, and shared water resources [102]. The prevalence of LSD across various countries, as shown in Figure 3, has raised serious concerns for the livestock industry. Although a few authorities regularly monitor LSD at a global scale, their constant disease–tracking efforts have failed to prevent the virus’s transboundary transmission [103]. As a result, it is critical that animal healthcare authorities, state governments, academics, and key stakeholders work together to provide active surveillance programs for the prompt detection of LSD outbreaks/clusters.

### 5.3. The Contribution of Wildlife to LSD Transmission

Seropositivity can indicate the potential involvement of animals in disease epidemiology, while mild clinical cases in wildlife may be overlooked due to difficulties in monitoring cutaneous lesions [103,104]. Previous research has demonstrated the susceptibility of impala, springbok, and giraffe to the virus [104,105]. Additional species that have tested positive for LSDV through serological testing include *Syncerus caffer* (African buffaloes), *Connochaetes taurinus* (blue wildebeest), *Taurotragus oryx* (eland), *Giraffa camelopardalis* (giraffe), *Aepyceros melampus* (impala), and *Tragelaphus strepsiceros* (greater kudu) [106,107]. In addition, LSD was documented in an *Arabian oryx* [108]; however, a precise understanding of the involvement of wildlife in LSD epidemiology remains limited.

## 6. Economic Impact

Lumpy skin disease (LSD) has had a profound economic impact on affected countries, leading to a substantial decline in milk production. The reduction in lactation output ranges from 10% to 85%, primarily due to increased body temperature and the subsequent development of mastitis [109]. Other consequences include damaged skin structures, decreased growth rates in cattle raised for meat production, temporary or permanent reproductive issues, spontaneous abortion, costs associated with medical treatments and vaccination, and mortality among affected animals [70,100,110,111]. Temporary reproductive difficulties may include reduced fertility, delayed oestrus, and infertility as a result of the fever and stress caused by the LSDV. In some circumstances, affected cows may experience transient changes in their reproductive cycles, resulting in missed breeding opportunities [70,100,110,111]. On the other hand, more serious consequences, such as chronic endometritis or other long–term damage to the reproductive system, might cause irreversible reproductive complications. In addition, LSD can cause orchitis (testicular inflammation) in bulls, which can result in long–term or permanent infertility [70,100,110,111]. These varied reproductive issues can significantly influence both short– and long–term cattle breeding programs and herd management, with reproductive issues affecting from 45% to 65% of cattle in industrial cattle farming [112]. A study found that LSD outbreaks in a herd of 393 animals in Turkey resulted in cumulative costs of 822,940.7 GBP [100,113]. In addition, in Ethiopia, losses were estimated at 6.43 USD per native zebu and 58 USD per Holstein Friesian cattle [102].

## 7. Prevention, Control, and Eradication of Diseases

The long–term stability of LSDV under ambient conditions has been scientifically proved. LSDV can survive in dehydrated skin lesions for a period of 25–50 days and can persist for several months in dark conditions within animal sheds [32]. Livestock managers need a thorough veterinary education to quickly diagnose LSD and minimize the risk of transmission [114]. Although the effectiveness of LSD treatment is debated, common strategies like symptomatic relief for inflammation, the use of antibiotics to prevent secondary infections and supportive therapies with a Vitamin B –complex and AD3E vitamins are commonly employed to enhance feeding and maintain reproductive health [5,115].

### 7.1. Diagnosis and Epidemiology

**Figure 4:**
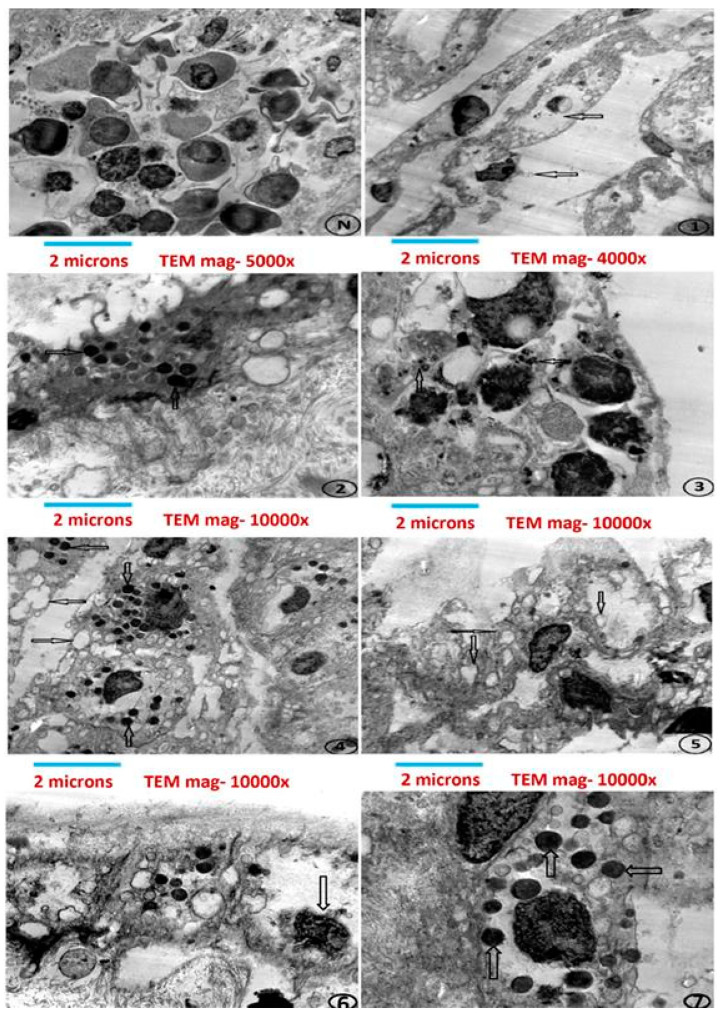
Electron micrographs of an inoculated chorioallantoic membrane (CAM) with a suspected Lumpy Skin Disease Virus (LSDV) sample, stained with uranyl acetate and lead citrate for enhanced visibility. The letter “N” indicates areas representing the negative control. The observed features include cell rupture, dark inclusion bodies, vacuoles, empty cells with distorted nuclei, and ovoid virus particles with rounded ends. Several key factors in these observations strongly suggest viral activity. Firstly, the presence of cell rupture and dark inclusion bodies indicates viral replication, which often disrupts host–cell structure. Additionally, the appearance of vacuoles and empty cells with distorted nuclei is commonly associated with viral cytopathic effects, as viruses typically manipulate host–cell machinery for replication, leading to such cellular abnormalities. Moreover, the identified ovoid particles with rounded ends match the morphological characteristics of known poxviruses, including LSDV. This specific shape and structure are less likely to be mistaken for components involved in the phagocytic pathway, which typically has a different cell morphology and particle formation. Therefore, by carefully analyzing these distinctive features, it is concluded that these are viral particles rather than artifacts of the phagocytic pathway. Further confirmation can be obtained through additional virological and molecular assays, which complement the electron microscopy’s findings. Reproduce under the Creative Commons Attribution License from reference [118]. Copyright 2022 BMC.

The diagnosis of LSD is typically made by correlating the clinical manifestations with laboratory findings that confirm the presence of the virus or its antigens [116,117]. Histological features are indicative of LSD and include nodules in subcutaneous tissue, all skin layers, and adjacent muscles, often accompanied by hemorrhage, necrosis, vasculitis, congestion, and edema [114]. Additionally, signs may include lymphocyte proliferation, thrombosis, perivascular fibroplasia, cellular infiltrates, infarction, and intracytoplasmic eosinophilic inclusions. In addition to conventional methods such as the histopathological examination of tissue samples and immunohistochemistry, alternative techniques for detecting LSDV include (a) virus isolation (b) electron microscopy [117,118], (c) immunofluorescence assay, (d) polymerase chain reaction (PCR), (e) agar gel immunodiffusion, (f) real–time PCR (qPCR), and (g) multiplex enzymatic immunosorbent tests [116,117]. A previous study investigated whether *Rhipicephalus (Boophilus) annulatus* ticks could transmit LSD among cows and buffaloes in Egypt. Samples were collected from potentially infected animals vaccinated with the Romanian SPPV [118]. The morphological identification and sequencing of partial cytochrome oxidase subunit I gene (COI) revealed a close relation to *R. annulatus*. Testing the ticks using the LSDV GPCR gene [118], along with transmission electron microscopy (TEM), provided insights into the virus’s characteristics (Figure 4) [118].

In addition, real–time polymerase chain reaction (qPCR) assays provide rapid and advanced means for detecting LSDV contaminants in the field settings, offering advantages over single or nested PCR approaches, in addition to a faster throughput and quantitative estimations [119,120]. Real–time PCR (qPCR) is an efficient method for confirming the presence of capripoxviruses like LSDV, with skin nodule samples showing positive results compared to blood or organ samples, which align with previous findings indicating the disease’s temporary presence in the bloodstream [120]. Similar confirmatory PCR results were obtained in Uganda to detect LSDV DNA in animals exhibiting skin nodules and clinical symptoms [104]. However, nucleotide sequence variations among different virus strains may introduce vulnerabilities, leading to discrepancies between the primer and target sequences, which may affect the amplification efficiency or yield negative PCR results [121].

### 7.2. Control Measures

Due to the viral nature of LSD, there is currently no targeted therapeutic intervention available [114,122]. The treatment approach for LSD primarily focuses on alleviating symptoms and using antibiotics to reduce the risk of potential bacterial complications. Antimicrobial agents, such as penicillin, cephalosporins, tetracyclines, and fluoroquinolones, are typically administered for a period of 5 to 7 days, with the duration of treatment depending on the severity of the condition [114,122]. Salib and Osmana have introduced a treatment strategy involving a combination of pharmacological agents targeting bacterial pathogens and inflammatory processes, which effectively mitigated the adverse effects of LSD and promoted survival by addressing infectious conditions [5]. They also suggested using antihistamines along with antipyretic medication containing paracetamol to reduce elevated body temperatures. To aid in the recovery from anorexia, a consistent regimen of multivitamins and medications supporting liver function was advised [5,123]. However, it is important to note that the therapeutic intervention for LSDV and its associated physiological impacts can incur significant costs and may not consistently result in a complete restoration of health [33]. Consequently, adopting a prevention strategy proves to be more effective in mitigating the substantial financial losses arising from hide impairment, reduced milk production due to mastitis, spontaneous abortion, elevated body temperature, fly larvae infestation, and mortality [33,122].

Upon the detection of LSD in a country or region, urgent measures such as halting movement and enforcing quarantine are essential. It is advisable to create zones in high–risk areas with limited movement and conduct clinical surveillance [124,125]. Furthermore, for the execution of eradication interventions, such as quarantine measures, the culling of infected and exposed animals, the appropriate disposal of carcasses, the thorough cleaning and disinfection of affected premises, and pest management, the timely identification of clinical signs during the outbreak is essential [125]. However, LSD can only be managed in regions in which it is prevalent through the implementation of vaccination, restrictions on animal movement, and the humane euthanization of the affected animals [87]. It is important to note that cattle hold a revered status in India, making euthanasia an impermissible practice [126]. Table 1 shows the therapeutic agents for the treatment of LSD.

#### 7.2.1. Vector Management

Managing vectors should be viewed as a supplementary strategy rather than a preventive one, as it cannot entirely stop the spread or transmission of LSDV to individuals. Implementing enhanced pest management techniques systematically, such as combining insecticides and pest repellents applicable to livestock, can aid in vector–control efforts within agricultural settings [33,84]. Additionally, there is an increased risk of LSDV transmission if diseased animals are not promptly removed [84].

#### 7.2.2. Vaccination

The optimal strategy to reduce lumpy skin disease transmission is through vaccinating cattle with a reliable vaccine, particularly when administered preventively or before the virus enters a susceptible area or country [130]. Live vaccines induce a robust and long–lasting immune response and have demonstrated to be effective in disease prevention [130]. Nevertheless, the administration of live vaccines can lead to localized inflammation and provoke a mild disease involving skin lesions [131]. Members of the Capripoxviruses have demonstrated protective effects in other contexts, helping to safeguard cattle against LSD [16,132]. In Ethiopia, the Gorgan GTPV vaccine effectively protects livestock from LSDV, while the Neethling and KSGP O–180 vaccines show limited efficacy, highlighting the need for additional molecular diagnostic methods to evaluate the vaccines’ effectiveness [132]. Contributing factors to vaccine failure include strain mismatch, issues with vaccine potency, and improper storage and administration. These factors suggest that the limited effectiveness of the Neethling vaccine in Ethiopia may be due to inadequate protection against diverse strains and reduced immunogenicity caused by attenuation [132].

Given the safety concerns surrounding live–attenuated LSDV vaccines, it is important for countries that have yet to use them to evaluate their potential role in protecting against sheep pox, particularly in regions free of LSD [87,133]. However, it is essential to note that the statement does not advocate for immediate vaccination against LSD in these LSD–free areas [87,133]. Instead, it highlights the need for the careful consideration of vaccination strategies in the context of local disease dynamics [87,133]. In addition, inactivated vaccines present a viable alternative, albeit at a higher cost and requiring multiple doses. These vaccines can be effectively combined with other antigens to develop polyvalent vaccines, which are advantageous in regions that may lack specific infections. After the initial use of live vaccines, inactivated vaccines can serve as a supplementary approach for disease control and eradication. Ultimately, the decision to adopt any vaccination strategy should be based on thorough risk assessments and the specific epidemiological context of each region [134]. For effective prevention against LSD, widespread, long–term immunization is crucial, especially before introducing new animals to affected farms. Calves of around three to four months, whose mothers have not been vaccinated or naturally infected, may have some immunity, while pregnant cows and breeding bulls should receive annual vaccinations [135].

Live attenuated vaccines are a type of immunization that contains weakened forms of pathogens that possess favorable immunogenicity and genetic traits, or attenuated strains that are carefully chosen and propagated under controlled conditions to maintain their immunogenicity [136,137]. However, live vaccines have some limitations, including adverse clinical reactions, the risk of reversion to a virulent form, and the possibility of emerging new diseases through homologous recombination with other viruses of the same genus [138,139]. Therefore, they are not recommended for use in regions in which the disease is absent. Additionally, immunosuppression is a crucial consideration following the administration of attenuated vaccines, as this can lead to a reduced immune response to the vaccine and increased susceptibility to secondary infections [140].

The Neethling strain, considered the prototypical strain of LSDV, was first identified as a virus genetically similar to vaccinia and was responsible for the Botswana 1943 and South Africa 1945 epidemics. Later, this strain was isolated and named Neethling [141,142]. A clinical study examined whether a live attenuated vaccine derived from the Neethling strain could serve as a preventive measure against LSD. However, the administration of this vaccine may result in mild adverse effects commonly known as Neethling disease [143]. Animals vaccinated with the Neethling–attenuated live strain vaccine exhibited a localized immune response, producing antibodies in cattle that lasted for over three years [143,144]. Notably, both vaccinated and non–vaccinated cattle demonstrated resistance to the virulent strain without a localized immune response due to several immunological mechanisms [143,144]. One explanation is the presence of pre–existing immunity, which may arise from prior exposure to related viruses or environmental strains that share similar antigens. This prior exposure can stimulate an immune response, enabling the immune system to recognize and combat the virulent strain even without vaccination. Additionally, the innate immune system provides an immediate, non–specific defense against pathogens through physical barriers, immune cells, and cytokines, which can help control or mitigate the severity of the infection before the adaptive immune response is fully activated [87]. Genetic variability among cattle may also play a role, as certain individuals may possess traits that enhance their ability to mount effective immune responses. Furthermore, environmental factors, such as overall health, stress levels, and management conditions, can influence immune responses; well–managed animals in optimal environments may exhibit stronger immune defenses compared to those in suboptimal conditions. Thus, while vaccination is a critical method for inducing targeted immunity, the interplay of previous exposures, innate immunity, genetic factors, and environmental conditions can contribute to the resistance observed in non–vaccinated cattle [87,145,146]. Moreover, recent research indicated that the Neethling vaccine reduced morbidity after vaccination [80]. The Neethling vaccine, developed and tested in Ethiopia, demonstrated limited efficacy in protecting vaccinated cattle against LSDV challenges during clinical trials, with a protection rate of only 30% [16]. A survey on a farm in northern Greece found that mature cows inoculated with the Neethling strain produced more milk than those that were not inoculated [144]. Approximately 12% of the vaccinated cows exhibited swelling, which subsequently resolved, and around 9% developed cutaneous nodules of less than 0.5 cm in diameter, while no nodules were observed in calves [144].

Vaccinated herds may experience a transient period of mild viremia. According to the European Food Safety Authority, the vaccination rate with the Neethling vaccine among cattle in Croatia was below 1% [147]. Recent research showed that the Ethiopian Neethling vaccination did not show any signs of protective effects against LSD, and the vaccine virus was detected in the milk of vaccinated cows [148]. Hence, a comprehensive assessment of the vaccine’s efficacy and safety is crucial to achieve the desired immunological impact. Multiple clinical trials evaluated and compared various homologous live attenuated vaccines for LSDV, including the Lumpyvax LSD Vaccine, Herbivac LS, Kenyavac, and the Marrokan Neethling O vivant LSD Vaccine. These vaccines can potentially induce a fever; however, no adverse effects on food consumption or overall well–being were observed across all experimental groups [149]. On the other hand, a study revealed that enlarged lymph nodes were observed in a group that received the Herbivac LS vaccine [133]. Following the Moroccan Neethling vaccination, animals exhibited smaller nodules compared to those of the infected animals [133].

A study found that the sheep and goat pox (KSGP) 0–180 strain vaccines developed in Kenya failed to protect cattle against LSDV. During an outbreak from 2006 to 2007, cattle vaccinated with Yugoslav RM65 showed clinical responses upon re–exposure to LSDV infection [16]. Another study suggested that the KSGP O–180 and KSGP O–240 strains might shed the vaccine strain, potentially infecting healthy, unvaccinated cattle [150]. Therefore, from 1989 to 2009, the Israeli government utilized the RM–65 vaccination strain to control LSD and sheeppox; however, the vaccine did not completely eliminate the disease [151,152]. Due to the random nature of attenuating mutations, it is likely that a single point mutation can eventually occur in animals, restoring virulence [17]. These unpredictable factors make attenuated vaccines a potential time bomb that could be triggered unexpectedly, leading to significant differences in the outcomes of certain vaccine strains between past and present centuries, possibly due to base pair mutations that occur during the manufacturing process [17]. The high frequency of recombination between identical DNA sequences in a virus’s double helix can reduce vaccine effectiveness, increasing viral pathogenicity post–vaccination and interactions with other viruses within the same family [153]. Therefore, when using live attenuated vaccines in humans, it is crucial to thoroughly investigate these issues and select more suitable vaccines based on the specific circumstances of the target livestock [17].

In contrast, the advantages of inactivated vaccines include their cost–effective production, quick development, and positive usage outcomes. Unlike live attenuated vaccines, however, inactivated vaccines usually require booster shots to maintain immunity and prevent viral infection [154]. In 1988, Blackall’s study showed that adjuvants can significantly enhance the effectiveness of inactivated vaccines [155]. Currently, there is no documented evidence of a commercially available inactivated LSD vaccine [17]. It was found that using Bi–ethylimine bromide to inactivate the attenuated Neethling strain also provides effective protection [134]. Various types of antibodies were identified, and the virus neutralization test indicated that the inactivated vaccine produced a 37% higher antibody response rate compared to the live attenuated vaccine by day 28 after immunization [156]. This vaccine successfully induced the production of substantial quantities of neutralizing antibodies, which is essential for animal welfare and compliance with animal ethics guidelines [156]. However, further experiments are necessary to confirm the specific clinical impact of this vaccine, as its efficacy has yet to be fully verified [156]. One limitation of inactivated vaccines is their ability to elicit a limited range of immune responses, which are effective in stimulating humoral immunity but less effective for mucosal and cell–mediated immune responses [17]. Thus, developing a safer and more optimized approach to targeting the lumpy skin disease virus is essential.

In contrast, live attenuated vaccines are advantageous because they retain all relevant antigens and allow for pathogen replication within the host, stimulating both cellular and humoral immunity. However, conventional techniques cannot fully attenuate all pathogens, and there is a risk of a reversion in virulence [17]. To address these challenges, researchers have focused on identifying the virulence genes of various pathogens and using targeted mutations or deletions and recombinant techniques to create attenuated strains [157]. Researchers developed a recombinant capripox virus vaccine using homologous recombination techniques to insert the rinderpest virus fusion protein gene after knocking out the thymidine kinase gene of the pox virus [158]. This approach successfully provided protection against rinderpest and reduced the risk of LSD in vaccinated cows [158].

A decade later, further research confirmed the reliability of rinderpest and goat pox recombinant vaccines [159]. A single vaccine dose protected cattle for up to one year, and some bovines demonstrated protection for up to three years. It was found that poxviruses can encode a protein similar to interferon gamma, which inhibits the binding of naturally produced interferon gamma to its receptor, allowing the virus to evade the immune response [159]. Another study revealed that the gene of the aphthous virus can encode a substance similar to interleukin–10, which induces immunosuppressive effects on the host’s cells [160,161]. Based on this premise, researchers used homologous recombination to delete open reading frames 005 and 008 of LSDV, developing recombinant vaccines that significantly enhanced neutralizing antibody production in vaccinated cattle. However, minor, temporary clinical reactions might occur during initial vaccination [162]. A previous study documented that LSDV–WB005KO provided protective effects against SPPV and GTPV in vaccinated animals, suggesting potential advantages for clinical application [163]. Regulatory authorities are concerned about the release of recombinant poxviruses and other vaccines into the environment due to perceived safety risks [17]. To address this, researchers have proposed investigating suicidal or non–replicating recombinant viruses. They pioneered replication–deficient adenoviruses, which can replicate in vitro within cells that possess the E1 region but cannot replicate in regular cells, making them non–hazardous even when exposed to healthy animals [164].

Advances in vaccine safety have led to a new approach to vaccine development through the recombination of LSDV with other viruses’ genes, such as parvoviruses, herpesviruses, adenoviruses, and retroviruses [17,87]. Due to these viruses’ large genomes, which range from 130 to 375 kb, these viruses can accommodate exogenous genetic material exceeding 25,000 nucleotide base pairs. Highly active promoters enable the simultaneous expression of multiple foreign genes, effectively activating both humoral and cellular immunity. Moreover, the virus has a limited host range and is considered safe as a vaccine carrier. Its thermal resistance potentially reduces the costs associated with refrigerated storage [17,87]. Additionally, a multivalent vaccine can prevent numerous diseases with a single administration, offering a safer and more cost–effective alternative to multiple injections of individual vaccines to achieve the same level of disease prevention [165,166].

In another study, the glycoprotein gene of the rabies virus was genetically integrated into the LSDV genome, creating a recombinant virus known as rLSDV–RG [167]. Clinical trials showed that this modified virus couldss trigger a strong humoral immune response [167]. The results indicated that 75% of the cattle showed significantly enhanced resistance to the rabies virus compared to the control group [167]. In a follow-up study, these findings were confirmed specifically in rabbits and mice, showing that vaccinated mice produced neutralizing antibodies at levels comparable to those seen with commercially available rabies vaccines [168]. Another study found that two recombinant viruses were created using LSDV as a vector, with the thymidine kinase gene of the lumpy skin disease virus serving as the insertion site for the glycoproteins of Bovine Epizootic Fever and Rift Valley Fever [169]. The LSDV–BEFV notably induced high levels of neutralizing antibodies, and mice vaccinated with LSDV–RVFV showed up to 100% protection against RVFV [169]. Therefore, this multivalent vaccination approach offers new methods and insights, potentially serving as a benchmark for developing recombinant LSDV–based–vaccines.

#### 7.2.3. Antiviral Treatment

Antiviral medications play a crucial role in combating viral infections by targeting various components of the virus or the host cell. These drugs specifically inhibit processes such as viral attachment, entry, uncoating, polymerase activity, and protease function, thereby disrupting the viral replication cycle [170]. However, the most promising options for antiviral treatment are natural compounds that demonstrate low cytotoxicity, high absorption, and significant efficacy [171]. The current management of LSD mainly consists of supportive care measures that include the application of wound care sprays, the administration of antibiotics to avert secondary infections, and the use of anti–inflammatory medications to alleviate pain and support appetite [172]. Recent studies have highlighted the potential of using viral protein inhibitors as effective antiviral agents. For instance, small molecule inhibitors have successfully targeted capsid proteins of enveloped viruses, disrupting essential protein interactions and thereby impeding viral replication [173]. In vitro studies have demonstrated that bee venom holds promise as an antiviral agent against LSDV. The venom not only inhibits viral activity but also preserves antigenic properties, indicating its potential as a therapeutic option for treating LSD [174]. Additionally, cellular protein kinase inhibitors have been shown to impede viral replication, positioning them as promising candidates for antiviral therapy. These inhibitors have proven effective against various viral infections by targeting crucial proteins involved in the viral life cycle [175].

Recent studies have revealed that LSDV induces endoplasmic reticulum (ER) stress, which activates the unfolded protein response (UPR) pathways. The activation of PERK and IRE1 signaling cascades is essential for sustaining LSDV replication, highlighting these pathways as potential targets for antiviral intervention [176]. Moreover, in vitro studies have shown that specific halogenated dihydrorugosaflavonoids possess significant antiviral activity against Chikungunya virus (CHIKV) by targeting non–structural proteins. This research provides a promising model that could potentially be adapted for the treatment of LSDV [177,178,179,180]. Recent advancements in antiviral research for LSD have underscored the potential of diverse therapeutic strategies, such as viral protein inhibitors, bee venom, and cellular protein kinase inhibitors. These discoveries pave the way for developing targeted treatments that address specific proteins involved in the LSDV life cycle, potentially leading to more effective management and control of the disease.

## 8. Conclusions and Prospects for Future Research

Buffaloes and cattle play a crucial role in the global economy; however, they are susceptible to LSD. This disease is characterized by distinct nodular lesions that primarily affect the dermal and subcutaneous tissues. Additionally, the conjunctiva, as well as the alimentary, urogenital, and respiratory systems, can also be sporadically involved, which poses a significant challenge for the livestock industry, as many nations are heavily reliant on agriculture. The lesions caused by LSDV have substantial economic impacts, including diminished hide quality, chronic illness, reduced milk production, weight loss, abortion, impaired fertility, and death. LSD can also lead to the decreased exportation of cattle and related livestock products. To accurately assess the prevalence of LSD, it is essential to investigate the factors contributing to its global spread, along with implementing epidemiological random screening across diverse geographical regions. The most effective approach to control the disease is through vaccination, combined with effective quarantine measures and vector control strategies. The recent spread of LSD into unaffected areas underscores its significant epidemiological and economic impact.

In endemic countries, veterinary officials need to closely monitor animal movements between countries due to the extensive boundaries. Combining thorough research on the disease’s epidemiology and transmission with proven preventative measures like vaccination can enhance the disease control and mitigation. Therefore, using accurate and rapid diagnostic techniques in high–prevalence areas, vaccination with a genetically similar strain of the LSDV, implementing vector control measures, restricting animal movement, and testing bulls involved in mating activities are all effective strategies to impede the transmission of LSD. However, control strategies must consider climatic and seasonal risk factors. Additionally, educating veterinarians, livestock workers, and herdsmen about LSDV transmission and management is crucial for mitigating the impact of LSD.

Developing advanced molecular diagnostic tools is crucial for the early detection and management of LSD in cattle herds. Enhancing current diagnostic methods to improve their sensitivity and specificity is necessary for prompt intervention and disease control. To achieve this, the research should focus on identifying novel biomarkers and genetic markers for more precise diagnostics. Additionally, there is a significant need to explore antiviral treatments that specifically target LSDV proteins. Investigating small molecule inhibitors, protein kinase inhibitors, and other antiviral agents that disrupt viral protein interactions could yield new therapeutic options. Evaluating these treatments’ effectiveness in vivo and developing safe formulations for cattle remains a priority. Furthermore, comprehensive studies on the interactions between LSDV and the host’s immune system will provide valuable insights into viral replication and pathogenesis. By understanding how LSDV evades the host’s immune responses, we can develop targeted therapies that bolster the cattle’s immune defenses against the virus.

While vaccination is a primary method for controlling LSD, the development of more effective and safer vaccines is needed. The research should aim to enhance the immunogenicity of existing vaccines, explore new vaccine candidates, and understand the long–term immunity they provide. Additionally, investigating vaccine delivery systems that ensure better coverage and protection across diverse cattle populations is essential. Extensive epidemiological studies are also critical for understanding the LSDV transmission dynamics. This includes identifying potential reservoirs, vectors, and environmental factors contributing to outbreaks. Through disease modeling, we can predict future outbreaks and assess the impact of various control strategies, ultimately aiding in the comprehensive management of LSD.

## Figures and Tables

**Table 1:** Therapeutic agents for the treatment of LSD.

No	Therapeutic Agents	Pharmacological Effects	References
1	Dexamethasone suspension	Anti–inflammatory steroids	[127]
2	Chlorpheniramine maleate	Antihistamine	[128]
3	Enrofloxacin	Antibiotic	[128]
4	Meloxicam	Anti–inflammatory nonsteroidal	[129]
5	Penicillin	Antibiotic	[127]
6	Tetracycline	Antibiotic	[127]
7	Oxytetracycline	Antibiotic	[129]

## Data Availability

No new data were created.
